# Modified Carboxyl-Terminated PAMAM Dendrimers as Great Cytocompatible Nano-Based Drug Delivery System

**DOI:** 10.3390/ijms20082016

**Published:** 2019-04-24

**Authors:** Minh Thanh Vu, Long Giang Bach, Duy Chinh Nguyen, Minh Nhat Ho, Ngoc Hoi Nguyen, Ngoc Quyen Tran, Dai Hai Nguyen, Cuu Khoa Nguyen, Thai Thanh Hoang Thi

**Affiliations:** 1 Institute of Chemistry and Materials, 17 Hoang Sam, Cau Giay, Hanoi 100000, Vietnam; vmthanh222@yahoo.com; 2 NTT Hi-Tech Institute, Nguyen Tat Thanh University, Ho Chi Minh City 700000, Vietnam; blgiang@ntt.edu.vn; 3 Center of Excellence for Functional Polymers and NanoEngineering, Nguyen Tat Thanh University, Ho Chi Minh City 700000, Vietnam; ndchinh@ntt.edu.vn; 4 Institute of Applied Materials Science, Vietnam Academy of Science and Technology, Ho Chi Minh City 700000, Vietnam; nhatho.dost@gmail.com (M.N.H.); hoi83bmt@gmail.com (N.H.N.); tnquyen979@gmail.com (N.Q.T.); nguyendaihai0511@gmail.com (D.H.N.); nckhoavnn@yahoo.com (C.K.N.); 5 Graduate University of Science and Technology, Vietnam Academy of Science and Technology, Hanoi 100000, Vietnam; 6 Biomaterials and Nanotechnology Research Group, Faculty of Applied Sciences, Ton Duc Thang University, Ho Chi Minh City 700000, Vietnam

**Keywords:** half generation polyamidoamine (PAMAM) dendrimer, carboplatin, PEGylation, drug delivery system, cancer treatment

## Abstract

Polyamidoamine (PAMAM) dendrimers are extensively researched as potential drug delivery system thanks to their desirable features such as controlled and stable structures, and ease of functionalization onto their surface active groups. However, there have been concerns about the toxicity of full generation dendrimers and risks of premature clearance from circulation, along with other physical drawbacks presented in previous formulations, including large particle sizes and low drug loading efficiency. In our study, carboxyl-terminated PAMAM dendrimer G3.5 was grafted with poly (ethylene glycol) methyl ether (mPEG) to be employed as a nano-based drug delivery system with great cytocompatibility for the delivery of carboplatin (CPT), a widely prescribed anticancer drug with strong side effects so that the drug will be effectively entrapped and not exhibit uncontrolled outflow from the open structure of unmodified PAMAM G3.5. The particles formed were spherical in shape and had the optimal size range (around 36 nm) that accommodates high drug entrapment efficiency. Surface charge was also determined to be almost neutral and the system was cytocompatible. In vitro release patterns over 24 h showed a prolonged CPT release compared to free drug, which correlated to the cytotoxicity assay on malignant cell lines showing the lack of anticancer effect of CPT/mPEG-G3.5 compared with CPT.

## 1. Introduction

Among the second generation of platinum-containing drugs, carboplatin (CPT) is known as the most important one that is widely used in clinics for the treatment of cancer [1]. It is clinically proven to combat numerous types of malignancies such as lung, ovarian, head and neck, endometrial, esophageal cancer, etc. [2]. After crossing the cell membrane and entering cells, CPT molecules are intracellularly activated by undergoing the hydrolysis of 1,1-cyclobutanedicarboxylate, becoming positively charged [3]. This allows CPT to form reactive platinum complexes that create inter-strand and intra-strand crosslinks with DNA and protein, thereby impeding DNA replication, transcription, and translation and suppressing proliferation [4]. In spite of having a similar molecular mechanism of action in cancer cells, CPT exhibits lower reactivity and toxicity compared to the first generation platinum-containing cisplatin, with no nephrotoxicity, ototoxicity, and neurotoxicity [5]. Moreover, it showed better effectiveness to several types of cancers that are resistant to cisplatin. This is thanks to the presence of CPT’s bidentate dicarboxylate as a replacement for labile chlorides of cisplatin [6,7]. However, myelosuppression, which causes the dramatic decrease in red blood cells, white blood cells, and platelets, is the main drawback of CPT [6]. Sometimes, the production levels of blood cells and platelets could be as low as 10% [8]. It also shown that a mean of 90% of administered CPT is excreted in urine within 24 h, and that CPT has an initial plasma half-life of 1.1 to 2 h [9,10]. To overcome these limitations, encapsulation of CPT in nanocarriers is a promising approach [1]. While most of the studies involved cisplatin, there are only a few investigations for CPT [11,12,13]. 

Polyamidoamine (PAMAM) dendrimer is a highly branched, globular nanostructure that has been greatly investigated for the delivery of drug molecules [14,15]. It possesses exceptional structural features including predetermined molecular weight, well-defined and stable structure, monodispersity, and high density of surface active groups that gives them the ease of functionalization [16,17,18]. Drugs can be encapsulated within PAMAM’s large internal cavity, entrapped on the surface, or interspersed throughout the dendritic structure, thereby protecting them from the physiological degradation [17,19,20,21]. Additionally, PAMAM’s nano-sized range is suitable for the passive targeting and accumulation of drug within the tumor site through enhanced permeation and retention (EPR) effect, thus reducing the side effects of loaded drugs [16,22]. Regardless of the many advantages, charge-associated toxicity limits the use of full generation amine-terminated PAMAM dendrimers. The electrostatic interaction between positively charged amino groups on the surface of PAMAM and negatively charged biological membranes leads to disruption of the lipid bilayer, causing cell lysis [23,24]. Moreover, positively charged amino groups also lead to the rapid clearance of amine-terminated PAMAM from blood circulation [14]. On the other hand, half generation PAMAM dendrimer with carboxylate groups on the surface does not interfere with cell membranes and is preferable for drug delivery. Some carboxyl-terminated PAMAM dendrimers were developed to deliver platinum-containing anticancer drugs [16,25,26,27,28,29]. Regarding CPT, Kang SJ and co-workers prepared PAMAM G3.5 loading CPT for murine retinoblastoma treatment. The formulation showed positive results with no associated toxicity. Nonetheless, the size range (>200 nm) and low drug loading capacity (47.54%) were undesirable [26]. In our previous works, carboxyl-terminated PAMAM dendrimers (G3.5 and G2.5) were prepared and utilized for cisplatin delivery. The size was effectively reduced and well-controlled, but the capacity for drug loading was dramatically decreased as well [16,25]. 

Polyethylene glycol (PEG) is a biocompatible, hydrophilic, and FDA-approved polymer that has gained great attention for the surface modification of PAMAM dendrimer [30,31,32]. Hydroxyl groups of PEG can be activated for the coupling reaction with PAMAM’s surface functional groups [14]. The conjugation of PEG onto PAMAM not only increases its cavity space for drug loading but also reduces the uncontrolled outflow of drug while traveling through the circulatory system and sustains the release of drug at target sites [14,33,34]. Non-specific interaction between serum proteins and PAMAM was also prevented by PEG, thus averting the uptake of PAMAM dendrimer by the reticuloendothelial system [35], reducing renal clearance, and improving the circulating half-life [36,37]. These favorable characteristics of PEG-conjugated PAMAM dendrimer were demonstrated in several studies [14,33,38,39,40]. 

In this study, carboxyl-terminated PAMAM generation 3.5 (G3.5) dendrimer was conjugated with poly (ethylene glycol) methyl ether (mPEG) and employed as a nanocarrier with great cytocompatibility for CPT delivery so that the drug will be effectively entrapped and not have uncontrolled outflow. CPT/mPEG-G3.5 is expected to have a suitable size that accommodates high drug loading efficiency. The chemical structure and morphology of the formulation were investigated. Further, live/dead staining and the resazurin cell viability assay were used to determine the ability of CPT/mPEG-G3.5 to minimize the toxicity of CPT. 

## 2. Results and Discussion

**Figure 1:**
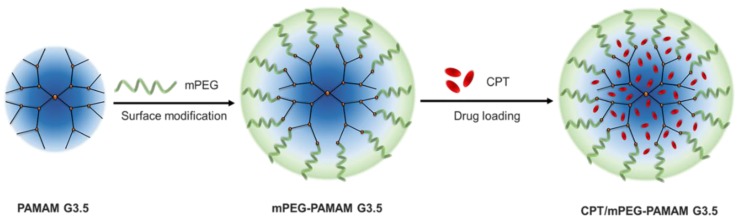
Illustration of carboplatin (CPT) encapsulated within mPEG-G3.5 dendrimer.

Therapeutic agents in chemotherapy are known to have side effects, ranging from mild to severe, in normal and non-targeted tissues without a proper delivery mechanism. Herein, PAMAM dendrimer, especially half-generation dendrimer, was chosen for its aforementioned advantages of pre-determined and controllable structure, presence of cavities and compartments for CPT encapsulation, and lack of positive charge-induced toxicity as in full generation dendrimer. Moreover, the carboxyl ends at the outermost of the half generation dendrimer are readily reactive to the amine-terminated mPEG, which is a widely used accessory molecule in drug delivery systems thanks to its comprehensive benefits (Figure 1). Theoretically, conjugation of mPEG can improve our system’s capacity of drug loaded and control pre-mature drug leakage from the particles while traveling to the target site, which are demonstrated through the following results.

**Figure 2:**
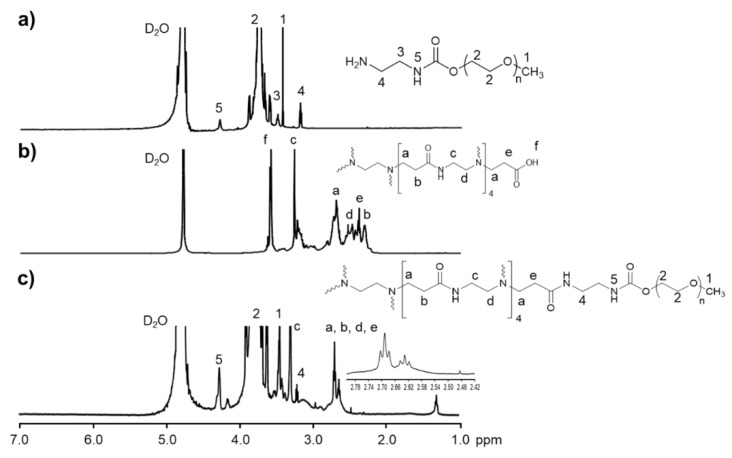
^1^H-NMR spectrum (D_2_O, δ in ppm) of (**a**) mPEG-NH_2_, (**b**) polyamidoamine (PAMAM) G3.5, and (**c**) mPEG-G3.5 dendrimer.

**Figure 3:**
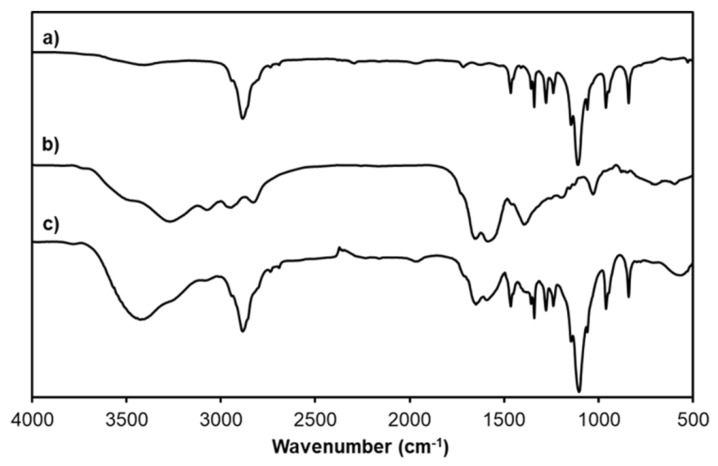
Fourier transformed infrared spectroscopy (FT-IR) spectra of (**a**) mPEG-NH_2_, (**b**) G3.5, and (**c**) mPEG-G3.5.

^1^H-NMR was used to analyze the chemical structure of the synthesized complex of PAMAM G3.5 and mPEG (mPEG-G3.5) (Figure 2). As shown in Figure 2c, signals at 2.78–2.84 ppm (a), 2.39-2.41 (b), 3.22–3.27 (c), 2.57–2.63 (d), and 3.59–3.60 (f) respectively assigned to N-CH_2_-CH_2_-, =N-CH_2_-CH_2_-CO, -CO-NH-CH_2_, CH_2_-N, and –CO-O-CH_3_ of PAMAM G3.5 were observed, which are similar to previous published studies [31]. The characteristic methyl and methylene protons at 3.44 ppm (1), 3.76 ppm (2), and 4.2 ppm (4) and a signal of N-H at 4.03-4.35 ppm (5) of mPEG-NH2 were clearly identified in the spectrum of mPEG-G3.5. The successful conjugation was shown by the absence of the resonance signals (f) at around 3.59–3.60 ppm in the spectrum of mPEG-G3.5 as compared to the spectrum of mPEG-NH_2_. The number of mPEG chains was experimentally determined by ^1^H-NMR based on the ratios of integral values for peaks assigned to mPEG (3.44 ppm) and dendrimer (3.22–3.27 ppm). Approximately 15 mPEG moieties were found to be attached to each PAMAM G3.5 dendrimer. The conjugation of PEG on the surface of G3.5 was further confirmed by Fourier transformed infrared spectroscopy (FT-IR) (Figure 3). Modified G3.5 possesses the same characteristic bands as G3.5, including the absorption at 3415–3265 cm^−1^ for -NH-, and at 1650 cm^−1^ for amide C=O, together with other absorptions at 1104 cm^−1^ of the C-O stretch and the C-H stretching at 2883 cm^−1^, which indicates the presence of mPEG in the synthesized complex.

GC analysis of blank run, references of ethylenediamine (EDA), MA, and methanol (MeOH), and mPEG-G3.5 were carried out. In the mPEG-G3.5 sample, diethyl ether as solvent forms the major peaks. Reference GC analysis detected the presence of MeOH, MA, and EDA at approximately the 3.0–3.1, 3.8, and 4.15-min marks on the retention timeline, which cannot be seen in the GC run of mPEG-G3.5. This result proved the purity of synthesized mPEG-G3.5, with nearly complete removal of excess EDA, MA, and MeOH solvent in the final product.

**Figure 4:**
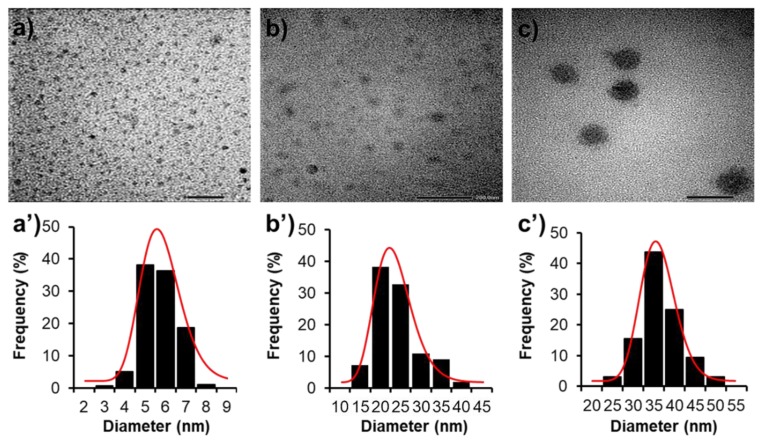
(**a**–**c**) Transmission electron microscopy micrographs and (**a’**–**c’**) size distribution of G3.5, mPEG-G3.5, and CPT/mPEG-G3.5 (respectively), fitted by log-normal distribution function (red line). Scale bar for a–c are 20, 200, and 50 nm, respectively.

The size and morphology of the unconjugated PAMAM, as well as the empty and loaded synthesized particles, were depicted in the TEM images (Figure 4). The fate of particles after being introduced into the body, including cellular uptake and targeting, has been proven to be size-dependent. The pharmacokinetics of the nanoparticles can be either enhanced or hindered with particles in different size ranges. Specifically, the enhanced permeability and retention (EPR) effect takes place within the nanoscale since the tumor capillary system recruited as tumor cells grow for sufficient supply of nutrients and oxygen usually lacks a complete endothelial lining, resulting in significantly higher permeability and hydraulic conductivity. However, the leakage rate from the vessels is quite slow, which means the particles must possess a long enough circulation time to sufficiently accumulate [41,42,43]. What is more, particles of size higher than 200 nm are often taken up selectively in the liver and spleen, and are likely to be cleared out of the circulation by the mononuclear phagocyte system (PMS) [31]. However, too small particles will be eliminated rapidly by either the kidneys or renal filtration, which both have an effective size cutoff at about 10 nm (i.e., particles less than 10 nm in size are subjected to elimination by these mechanisms) [44]. Post-modification G3.5 particles showed a clear increase in size, from 5.68 ± 0.06 nm to 22.7 ± 0.4 nm, which visibly confirms the successful conjugation of mPEG onto the surface of PAMAM. After loading of CPT, the particle size saw another increase to 36.0 ± 0.2 nm, which falls into the optimal size range for nanocarriers. This indicates an improvement in controlling the size of dendrimer particles, as previous study also on PEG-modified G3.5 resulted in size that was bigger than desired [26], while another one resulted in size that was too small [25]. The conjugation of mPEG, therefore, can be utilized to control the size of the dendrimer particles. Moreover, particles at this size can also be expected to have prolonged circulation time thanks to bypassing both PMS and kidney or renal clearance, ensuring a therapeutic level of drug to build up at the tumor site by EPR effect.

**Figure 5:**
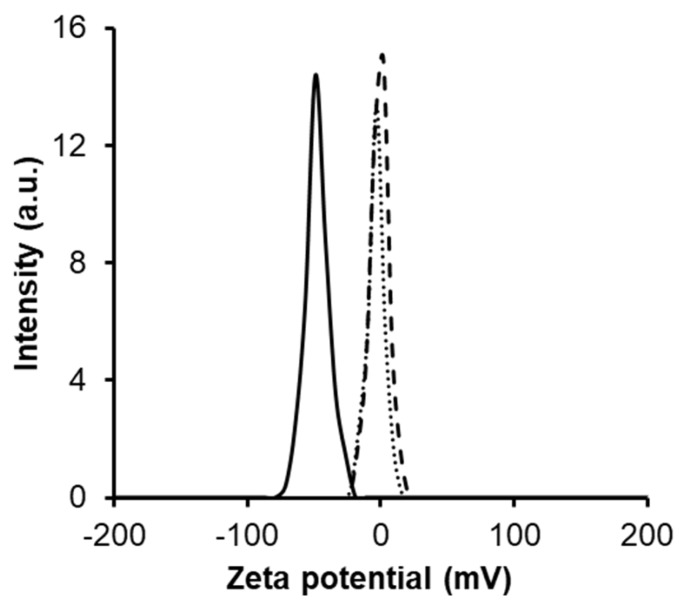
Zeta potential of PAMAM G3.5 (solid line), mPEG-G3.5 (dashed line), and CPT/mPEG-G3.5 (dotted line).

Apart from particle size, surface charge is another parameter frequently used in characterizing of nanocarriers. Cationic charged particles have been found triggering non-specific interactions with cell membranes or opsonizing proteins owing to electrostatic bindings, which can result in unexpected cytotoxicity [31]. Moreover, various in vivo studies have associated more negative zeta potential with higher clearance rate from the blood, possibly also due to undesirable bindings with opsonizing proteins [45,46,47]. Thus, less negatively charged or almost neutral particles may be considered more rational [48]. Figure 5 illustrates the zeta potentials of the proposed particles, uncoated, PEG-coated, and CPT-loaded. As can be seen, naked PAMAM G3.5 had a deeply negative charge of −50.07 mV, which was to be expected since half generated dendrimers are coated with negatively charged carboxylate groups. However, after being coated with PEG, the surface charge increased to nearly neutral (1.49 mV). The reason for this increase could be due to the capping of PEG over the carboxylate groups, which in a sense further proves the conjugation of PEG. The zeta potential of the final product, CPT/mPEG-G3.5, did not show any significant difference from the previous value (1.32 mV). The near to neutral surface charge not only lessens the chance of toxicity caused by high cationic charge but also prevents premature clearance of the carriers.

Drug loading efficacy is considered to be essential in the design of any drug delivery system, since it relates more or less directly to the therapeutic activity of the system. In our study, the DLE of the proposed system reached 85.37% ± 7.87%, which was exceptionally high compared to previous studies on a similar system of G3.5 but without mPEG conjugation [26]. Therefore, it is conclusive that the mPEG on the surface of the particles can play a significant role in providing more space for drug loading, and also capping the cavities to keep in the drug after loading.

**Figure 6:**
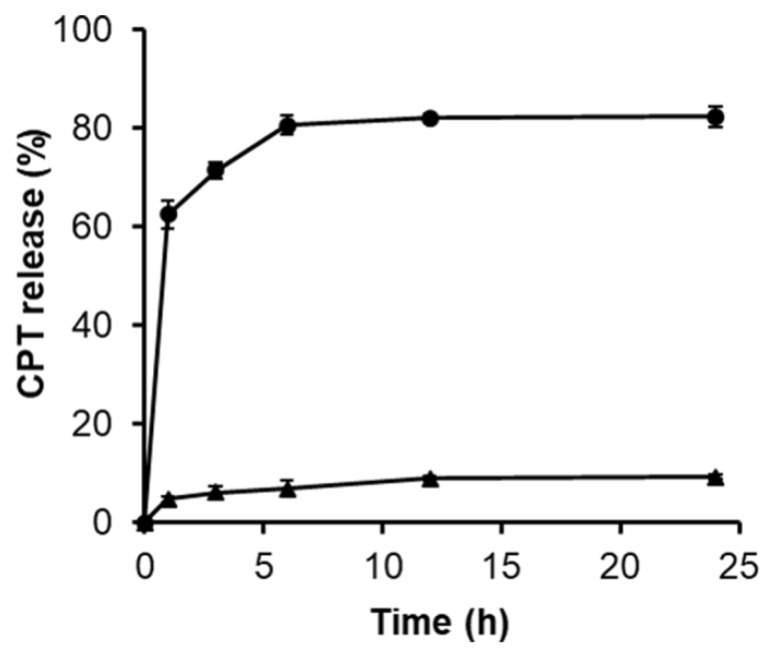
In vitro release profiles over the course of 24 h of free CPT (round) and CPT from mPEG-G3.5 (triangle).

As shown in the drug release profiles of free CPT and loaded CPT (Figure 6), a burst release up to more than 60% of the free drug was observed in the first hour, whereas the loaded system showed a much more prolonged behavior. This could be seen as an improvement in comparison to our prior formulation, which experienced more rapid release [22]. However, this formulation still has room to improve, since the accumulated drug released after a longer period of time should display a gradual rise to be controlled. Yet, a sustainable release of CPT is essentially desirable, as CPT has a very short initial plasma half-life (only up to 2 h) [6], and is rapidly eliminated through urination. Moreover, after administration, the particles together with their cargos need time to reach their target sites, not to mention that in the case of tumor passive targeting, the EPR effect also takes time to accumulate, due to the low leakage rate as mentioned above. During this time, preventing unwanted outflow of drug out of the delivery particles is necessarily important, not only to minimalize side effects to untargeted tissue but also to ensure the amount of drug reaching the target site. Therefore, the design of an antitumor agent delivery system needs to demonstrate an initial non-leakage time.

**Figure 7:**
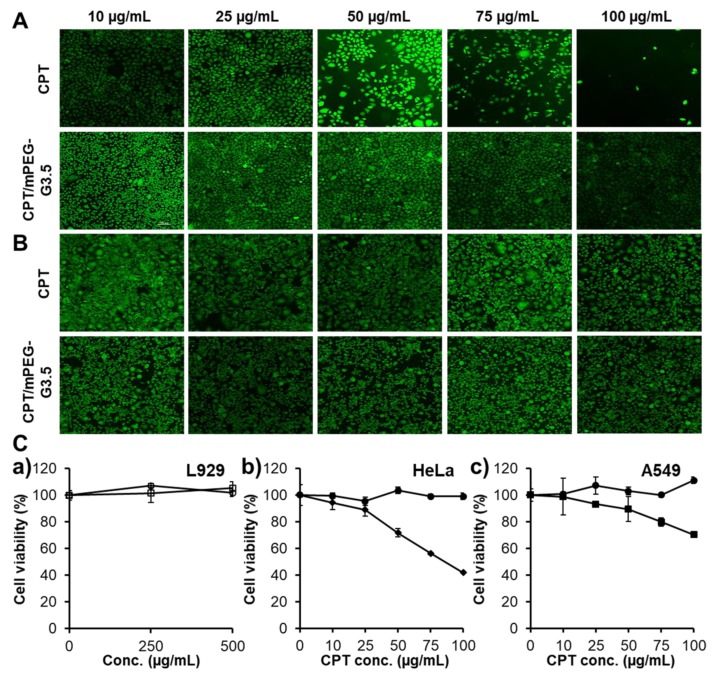
Cell viability after 24 h in percentage and fluorescent signals of HeLa (**A**,**C**-**b**) and A549 (**B**,**C**-**c**) with CPT (square) and CPT/mPEG-G3.5-CPT (round); Cell viability in percentage after 24 h of L929 (**C**-**a**) with mPEG-G3.5 (white dot) and CPT/mPEG-G3.5 (black dot)

The cytotoxicity of our synthesized system, empty and loaded (mPEG-G3.5 and CPT/mPEG-G3.5) towards normal cells was demonstrated by resazurin assay with normal fibroblasts (L292 line) (Figure 7C-a). As can be seen, up to the concentration of 500 µg/mL for 48 h, the system showed no toxicity to normal cells, which proves the safety of mPEG-G3.5 systems at the tested concentration. Cell viability assay results of free CPT and CPT/mPEG-G3.5 particles on two malignant cell lines, HeLa and A549, were illustrated by live/dead assay (Figure 6B and Figure 7A) and resazurin assay (Figure 7C-b,c). Within the course of 24 h, free CPT established restricted toxicity towards HeLa and A549 cell lines, with a clear concentration-dependent tendency, while CPT/mPEG-G3.5 has not shown significant toxicity. This can be accounted for by two possible explanations: the mechanism of CPT itself and the release profile of CPT/mPEG-G3.5 [18]. CPT exerts toxicity on cells through binding with nuclear DNA forming DNA adducts, including mono- and di-adducts, and inducing apoptosis. However, it is generally accepted that the 1,2-intrastrand di-adduct is responsible for this antitumor effect, due to the fact that it is specifically recognized by High Mobility Group proteins, and less effectively repaired through nucleotide excision [49]. A previous study showed that mono-adducts accumulate in nuclear DNA over 2 days at least, due to a slow diffusion of CPT to nuclear DNA in cells [50], which may interfere with the cytotoxicity rate of both free CPT and encapsulated CPT in our assay, as lower concentrations of drug may establish a lower diffusive gradient, and thus require more time for sufficient accumulation in nuclear DNA. Moreover, as the above release profile implies, the amount of accumulated CPT after 24 h of release also corresponds to the low death rate in the first 24 h. Further in vitro and in vivo research should be carried out in future studies to gain more detail about the therapeutic efficacy of developed formulations.

## 3. Materials and Methods

### 3.1. Materials

Carboplatin (CPT, Mw 371.26 Da) was purchased from TCI (Tokyo, Japan). Ethylenediamine (EDA) and toluene were purchased from Merck (Darmstadt, Germany). Methyl acrylate (MA, Mw 86.09 Da), 4-Nitrophenyl chloroformate (NPC, Mw 201.56 Da), poly(ethylene glycol) methyl ether (mPEG, Mw: 5 kDa), tetrahydrofuran (THF), 1-ethyl-3-(3-dimethylaminopropyl)carbodiimide (EDC), and diethyl ether were supplied by Sigma-Aldrich (St. Louis, MO, USA). Methanol was received from Fisher Scientific. Spectra/Por^®®^ Dialysis Membrane (MWCO 3.5 kDa, 6–8 kDa, and 12–14 kDa) was purchased from Spectrum Laboratories, Inc. (Rancho Dominguez, CA, USA). All reagents and solvents were used as received without further purification.

### 3.2. Synthesis of Carboxyl-Terminated PAMAM G3.5 Dendrimer

**Scheme 1:**
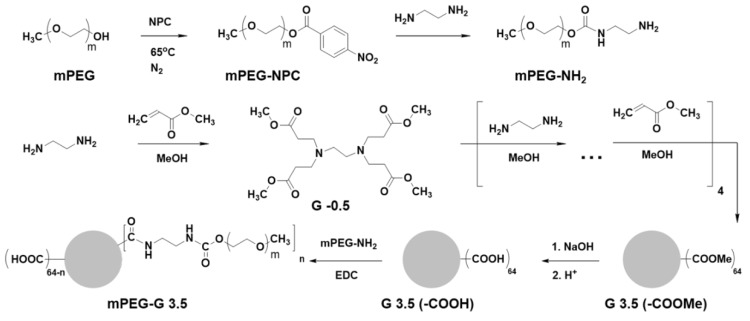
Synthetic scheme of amine-terminated poly (ethylene glycol) methyl ether (mPEG) and mPEG-G3.5 dendrimers. The terminal hydroxyl group of mPEG was converted to a primary amine by using 4-Nitrophenyl chloroformate (NPC), followed by the reaction with ethylenediamine (EDA). Amine-terminated mPEG was attached onto G3.5 dendrimer, utilizing 1-ethyl-3-(3-dimethylaminopropyl)carbodiimide (EDC) as a carboxyl activating agent.

Half-generation carboxyl-terminated PAMAM G3.5 was synthesized from the EDA core by a divergent approach that employs two consecutive chain-forming reactions, including Michael addition reaction and amidation reaction, as reported previously by Donald Tomalia with minor modification (Scheme 1) [31]. The Michael addition reaction between primary amine groups of EDA and excess acrylate groups of MA gives the half generation carboxyl-terminated PAMAM denoted by Gn.5, followed by the amidation reaction between methyl propionate groups of carboxyl-terminated PAMAM with excess EDA to generate full generation amine-terminated PAMAM, designated Gn. Shortly, 20 mL of EDA was added to 150 mL of MA dissolved in methanol and kept under constant stirring (3 h at 0 °C and then 2 days at room temperature). Solvent and unreacted starting materials were discarded by rotary vacuum evaporator (Strike 300, Lancashire, PR6 0RA, UK) to collect the core precursor G-0.5. After that, a slow addition of G-0.5 (20 g in 10 mL methanol) to 130 mL EDA solution was performed to obtain PAMAM G0.0. The mixture was stirred at room temperature for 4 days, rotated under vacuum using mixed solvent toluene: methanol (9:1 *v*/*v*), and dialyzed by dialysis membrane (MWCO 3.5 kDa) against methanol to eliminate excess EDA and toluene. Finally, methanol was removed from the obtained product by drying under vacuum. These two reactions reiterated continuously to give the next higher generation of PAMAM dendrimer.

### 3.3. Amination of mPEG

Terminal hydroxyl group of mPEG was converted to primary amine using the following procedure. Firstly, NPC (0.4837 g, 2.4 mmol) was added to molten mPEG (10 g, 2 mmol) at 65 °C and stirred for 5 h under nitrogen atmosphere. The mixture was cooled down to 40 °C, followed by the addition of 15 mL of THF and then maintaining the constant stirring for 12 h at room temperature. Next, the obtained solution was added dropwise into diethyl ether for the precipitation, filtered, and dried under vacuum to achieve the powdery form of activated mPEG-NPC. Thereafter, mPEG-NPC (7 g, 1.355 mmol) dissolved in distilled water was added dropwise to EDA solution (1.8 mL). The mixture was stirred at room temperature for 24 h. Finally, the resulting solution was dialyzed against distilled water (dialysis membrane MWCO 3.5 kDa) for 3 days and lyophilized to give amine-terminated mPEG.

### 3.4. Conjugation of mPEG to PAMAM G3.5 (mPEG-G3.5)

For the conjugation of mPEG to PAMAM G3.5, synthesized PAMAM G3.5 was mixed with EDC 5% and then added into mPEG-NH_2_ (6.0 g) prepared in distilled water. After 24 h of stirring at room temperature, the resulting solution was put into a dialysis bag (MWCO 12–14 kDa) and dialyzed for 3 days against distilled water to eliminate the impurities. Finally, purified product was lyophilized to obtain powder form for future use.

### 3.5. Characterizations

FT-IR/NIR Spectroscopy Frontier (Perkin Elmer, Waltham, MA, USA) and Bruker AC 500 MHz (Bruker Co., Billerica, MA, USA) were used to analyze the chemical structure of synthesized products. To obtain the FT-IR spectra, samples (1–2 mg) were prepared by mixing them with dried KBr (100–200 mg) and then pressing the mixture into a pellet. The measurement acquired in range of 4000–500 cm^−1^ with a resolution of 4 cm^−1^. Gas chromatography (GC) (PerkinElmer Clarus 680) analysis of final product was carried out. Synthesized mPEG-G3.5 was dissolved in diethyl ether as solvent for GC run. TEM (JEM-1400, Tokyo, Japan) with an accelerating voltage of 100 kV was used to depict the size and morphology of the products. Sample at a concentration of 1 mg/mL was placed on a carbon-copper grid (300-mesh, Ted Pella Inc., Redding, CA, USA) and air-dried for 10 min before the measurement. Zeta potential and hydrodynamic diameter of the products were determined by Nano Particle Size SZ-100 (Horiba, Kyoto, Japan). Samples were prepared with deionized water at a concentration 1 mg/mL, filtered (pore size 0.45 μm), and sonicated for 5 min. Each sample was measured 3 times.

### 3.6. Preparation of CPT/mPEG-G3.5 and Drug Loading Capacity

Briefly, 10 mg of CPT was dissolved in methanol, kept under constant stirring for 1 h to completely dissolve the drug, and added slowly to the 10 mL of mPEG-G3.5. The mixture was carried out at room temperature for 24 h, followed by the removal of solvent under vacuum. The resulting mixture was then put into the dialysis bag (MWCO 3.5 kDa) for the dialysis against deionized water in 1 h. The dialyzed solution was freeze-dried to yield the product in powder form while the total volume of solution outside the dialysis bag that contains unloaded CPT was collected to determine the drug entrapment efficiency. The entrapment efficiency (%) of CPT/mPEG-G3.5 was determined by the high performance liquid chromatography (HPLC) method regarding the initial fed CPT and loaded CPT, which was calculated indirectly from the unloaded CPT.

### 3.7. In Vitro Release Study

For the in vitro CPT release study, a 1 mL suspension of CPT/mPEG-G3.5 (CPT content, 0.2 mg/mL) in phosphate buffer medium was loaded into a dialysis bag (MWCO 3.5 kDa). The dialysis bag was immersed into a vial containing 14 mL of PBS (0.01 M, pH 7.4), followed by placing the vials in an orbital shaker bath for temperature maintaining (37 °C) and continuously shaking (100 rpm). At predetermined time intervals, the release medium (14 mL) was collected, filtered (pore size = 0.22 μm), and replaced with an equivalent volume of fresh media. After lyophilizing the collected media, the amount of released CPT was measured by HPLC (Prominenece LC-20A, Shimadzu, Kyoto, Japan).

### 3.8. Cell Viability Tests

Resazurin assay was performed to investigate the cell viability of synthesized products against HeLa cells, MCF-7 cells, and normal mouse fibroblast cells L929. Cells (1.5 × 105 cells/well) were seeded in a 96-well plate containing 100 μL of Dulbecco’s Modified Eagle’s medium (DMEM, Gibco, Invitrogen) supplemented with 10% fetal bovine serum and 1% penicillin/streptomycin and cultured for 1 day under humidified atmosphere (98%) containing 5% CO_2_, at 37 °C. Then, the media were discarded and replaced with fresh media containing free CPT (10–100 μg/mL) or CPT/mPEG-G3.5 dendrimer containing equivalent CPT concentration for the next 24 h of incubation. In control wells, cells were treated with medium only and assigned to 100% survival. After removing the supernatant, cells were washed twice with PBS, treated with resazurin (10 μL, 0.2 mg/mL), and incubated for 4 h. The fluorescent signal was detected in a micro-plate reader (Varioskan™ LUX, Thermo Scientific, Waltham, MA, USA) at Ex/Em 560/590 nm. The relative cell viability was calculated by normalizing the fluorescence intensity of samples to that of control group as following equation:

(1) $$\mathrm{Cell}\;\mathrm{viability}\;\left(\%\right)=\frac{\left({\left[\mathrm{Abs}\right]}_{\mathrm{sample}}-\;{\left[\mathrm{Abs}\right]}_{\mathrm{blank}}\right)}{\left({\left[\mathrm{Abs}\right]}_{\mathrm{control}}-\;{\left[\mathrm{Abs}\right]}_{\mathrm{blank}}\right)}\;\times \;100$$

In addition to the resazurin assay, cell viability assessment of the products against HeLa and MCF-7 cells was visualized by live/dead staining. In short, cells in each well were mixed with 25 μL of fluorescein diacetate (FDA) (10 mM) and ethidium bromide (EB) (7.5 mM). After 3 min of incubation, cells were rinsed several times with PBS. The results were observed using microscope (Eclipse Ti-E Inverted Microscope System, Nikon, Tokyo, Japan) at excitation wavelength of 485 nm for FDA and 530 nm for EB.

## 4. Conclusions

Spherical mPEG-G3.5 was successfully prepared with the size within the optimal range for nanocarriers and nearly neutral surface charge. The drug loading efficiency was highly improved, especially the entrapment efficiency, reaching up to more than 80%. Cell viability assay results with three cell lines correspond with the sustained release profile of the prepared particles. Even though mPEG-G3.5 has great cytocompatibility, the lack of anticancer effect of CPT/mPEG-G3.5 compared with CPT on tested malignant cell lines implied that CPT/mPEG-G3.5 is not an appropriate solution as anticancer therapy.
